# Mycobiota and Antifungal Antibodies as Emerging Targets for the Diagnosis and Prognosis of Human Diseases

**DOI:** 10.3390/jof11040296

**Published:** 2025-04-09

**Authors:** Eguzkiñe Diez-Martin, Leidi Hernandez-Suarez, Egoitz Astigarraga, Andoni Ramirez-Garcia, Gabriel Barreda-Gómez

**Affiliations:** 1 Research and Development Department, IMG Pharma Biotech S.L., 48170 Zamudio, Spain; 2 Department of Immunology, Microbiology and Parasitology, Faculty of Science and Technology, University of the Basque Country (UPV/EHU), 48940 Leioa, Spain

**Keywords:** mycobiota, antifungal antibodies, commensal fungi, pathogenic fungi

## Abstract

The human body is colonized by diverse microorganisms, with bacteria being the most extensively studied. However, fungi, collectively known as “the mycobiota,” are increasingly recognized as integral components of the microbiota, inhabiting nearly all mucosal surfaces. Commensal fungi influence host immunity similarly to bacteria and contribute to other essential functions, including metabolism. This emerging understanding positions fungi as potential biomarkers for the diagnosis and prognosis of various diseases. In this review, we explore the dual roles of fungi as both commensals and pathogens, and the potential of antifungal antibodies to serve as diagnostic and prognostic tools, especially in chronic immune-inflammatory non-communicable diseases, including inflammatory bowel disease, rheumatoid arthritis, multiple sclerosis, and neurodegenerative disorders. Finally, we address current challenges and outline future perspectives for leveraging fungal biomarkers in clinical practice.

## 1. Fungal Microorganisms: Their Place in the Human Body

The human body is not solely composed of human cells, it also hosts a vast number of microbial cells known as “the microbiota,” including viruses, archaea, bacteria, fungi, and protozoa [1]. The microbiota lives in symbiosis with the human host, contributing to health by stimulating the immune system, protecting against pathogens, and aiding metabolism and neurologic activity, among other functions. However, when the balance of the microbiota is disrupted—a state known as dysbiosis, characterized by alterations in commensal communities, including their loss or imbalance—physiological processes can become dysregulated, and opportunistic or pathogenic microorganisms may colonize, potentially leading to disease [1,2,3,4]. It should be noted that the term “microbiota” encompasses both commensal and pathogenic microorganisms that live in and on a multicellular organism [5].

While bacteria have traditionally been the most extensively studied component of the microbiota, fungi form an equally important group, collectively referred to as “the mycobiota” [1,3,6]. Despite being fewer in number, representing approximately 0.1% of the total microbial population in the gut, fungi are approximately 100 times larger than bacteria, allowing them to occupy significant physical space, and they possess a unique metabolism, enabling them to play critical roles in both maintaining health and contributing to disease [1,6,7].

This review examines the essential role of fungal communities in humans, the mechanisms by which the immune system combats fungal infections, with a particular focus on the humoral response, and explores how this immune response can be leveraged for clinical purposes, including its use as both a diagnostic and prognostic tool. Finally, this review will conclude by discussing the limitations and challenges of using antifungal antibodies as a diagnostic tool, as well as future perspectives.

## 2. Fungal Communities in the Human Organism

Inside the human body, fungal communities (a collection of metabolically active fungi that coexist in the same environment and either interact or have the potential to influence one another) may be grouped depending on their relationship with the host into commensal species, which support health and homeostasis, and pathogenic species, which trigger diseases.

### 2.1. The Process of Fungal Commensalisms

**Figure 1:**
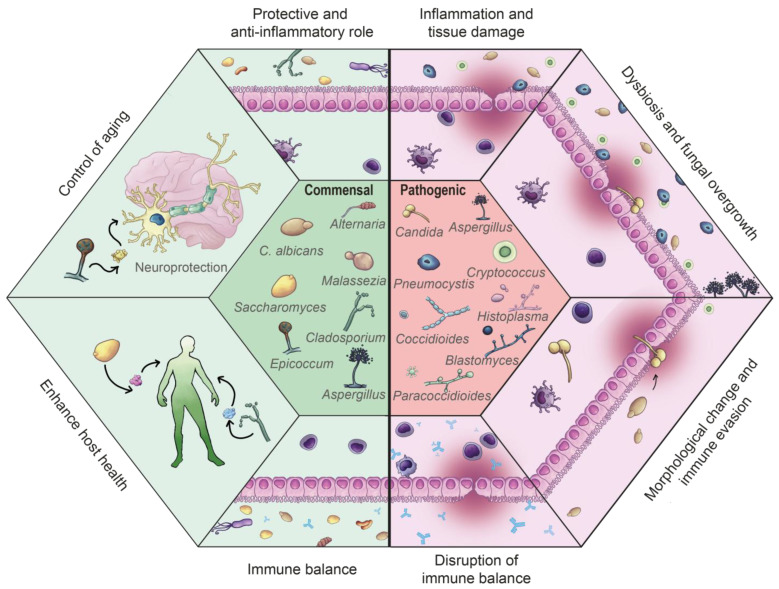
Main commensal and pathogenic fungal species in the human organism and their functions.

Commensal microorganisms are those that form a relationship with the host in which they do not cause any damage, having no negative impact on the host’s fitness, and sometimes even can play beneficial roles (Figure 1). Nevertheless, the definition of commensalism is not always straightforward, as these interactions exist along a continuum and can shift from pure commensalism to mutualism or even parasitism, depending on various factors, including environmental conditions [8,9,10,11].

Specifically, fungi in the microbiota interact with the host’s immune system, helping to maintain immune balance and possibly affecting the onset and progression of immune-related diseases. This relationship can be intricate and varies depending on the context, as some fungi may encourage tolerance while others can heighten inflammation. They also impact the control of aging [12], providing bioactive compounds, such as ergothioneine, that support neuroprotection and longevity. Additionally, they produce both primary and secondary metabolites that affect the host’s metabolism and physiology, including how nutrients are processed and metabolic pathways are regulated. Some fungal metabolites exhibit antimicrobial and anti-inflammatory properties, which can modulate bacterial populations and influence the host’s inflammatory responses. Furthermore, fungi participate in complex relationships with other microbial communities, especially bacteria, within the host. These interactions can greatly impact the overall composition and function of the microbiota, ultimately influencing disease status and treatment outcomes [5,12,13,14,15,16]. In fact, they are so important that increasing evidence suggests a correlation between diseases and the mycobiota [17]. As a result, to promote a healthier mycobiota alongside a balanced microbiota, various therapies have been developed over the past few decades, including probiotics, prebiotics, fecal microbiota transplants (FMT), dietary interventions, and antifungal metabolites [13,18,19,20].

Fungi can be found throughout the human body, including on the skin and in the mucosa of the gastrointestinal, respiratory, and genitourinary tracts—particularly in the female reproductive tract, where sex hormones influences fungal colonization [2,3,14,17,21]. Mucosal surfaces are particularly important, as fungi are commonly present on the mucosa in most humans, making these surfaces the primary entry point for many fungal infections. For example, *Candida albicans*, a common fungus on mucosal surfaces, can transition from harmless commensals to opportunistic pathogens under certain conditions, such as microbiota imbalance, barrier disruptions, or immune dysfunction [22,23].

Dominant fungal genera in healthy individuals include *Candida* spp., *Malassezia* spp., *Aspergillus* spp., *Epicoccum* spp., *Saccharomyces* spp., *Alternaria* spp., and *Cladosporium* spp., although their presence and abundance may vary significantly between specific individuals as they are influenced by several factors [5,17]. Their presence begins at birth, with composition influenced by factors such as delivery mode (vaginal or cesarean), maternal exposure to probiotics or antibiotic prophylaxis, gestational age, infant feeding practices, maternal diet, environment, and host genetics. After birth, this initial fungal composition undergoes continuous change and development throughout the host’s life, influenced by many factors, including health status and underlying disease, environment, diet and medication use, and the seasons [14,24].

### 2.2. The Process of Fungal Infection

Similar to the bacterial microbiota, many inflammatory diseases of non-infectious origin are the result of alterations in the mycobiota. The invasion of exogenous fungi may disrupt the fungal microbiota, leading to the colonization of opportunistic and pathogenic fungi, which in turn triggers negative impacts on health [1,3,14,17]. Dysbiosis could also occur first, leading to colonization by harmful fungi, with the same negative host health consequences [25].

However, fungal diseases are relatively rare, as only around 300 species of fungi, mainly belonging to the genera *Aspergillus* spp., *Candida* spp., *Cryptococcus* spp., and *Pneumocystis* spp., are known to cause human diseases. Although infections with these fungi can occur more frequently, the transition to disease, which disrupts homeostasis and causes damage, is particularly concerning due to case fatality rates that can reach up to 90% [26,27,28].

The rarity of fungal infections in humans is thought to be due to the complex defense mechanisms that mammals have developed through co-evolution with fungi. For instance, human body temperature is too high for most fungi, physiological fluids have a slightly alkaline pH unfavorable for fungal growth, and the immune system plays a crucial role in preventing fungal infections. Consequently, these infections primarily occur in individuals with underlying conditions that compromise immunity, such as HIV infection or hematologic malignancies, or in those experiencing microbiota disruption [25,29,30].

Nevertheless, a significant group of fungi for clinical settings, the dimorphic pathogens, can cause severe infections even in immunocompetent individuals. These fungi, including genera like *Coccidioides* spp., *Histoplasma* spp., *Paracoccidioides* spp., and *Blastomyces* spp., possess the unique ability to switch between hyphal and yeast forms, a key factor in their pathogenicity. Notably, this morphological plasticity, specifically the yeast-to-hypha transition, has been independently described in multiple saprophytic fungi, highlighting its evolutionary significance beyond pathogenesis [29,31].

Certain fungal genera, such as *Candida* and *Aspergillus*, exhibit a dual role, functioning as both commensals and opportunistic pathogens. This characteristic is central to their biology. In opportunistic fungi, this transition from commensal to pathogenic is a complex process influenced by various factors, such as host conditions, environmental changes, fungal gene expression modification and immune evasion, and the previously mentioned microbiota dysbiosis. Notably, these fungi, while existing as commensals in healthy individuals, possess the potential to become pathogenic when environmental or host-related factors—such as immune suppression, microbiota imbalance, or changes in nutrient availability—disrupt microbial homeostasis. *C. albicans*, as the archetypical opportunistic pathogen, serves as a key model for studying this transition. Among host-related factors, the integrity of defensive barriers and the functionality immune system play pivotal roles. Upon the fungus dissemination, it encounters significant environmental changes, such as shifts in oxygen levels, nutrient availability, and pH, prompting adaptations through regulatory networks that modulate gene expression. These adaptations enable the fungus to thrive in diverse conditions and prepare for infection, including morphological changes, such as the yeast-to-hyphal transition, which is a crucial step for the full virulence of *C. albicans* [22], and the development of mechanisms to evade both humoral and cellular components of the host immune system, for example, by the production of pore-forming proteins to evade the phagocytosis [32], enzymes that degrade complement system [33,34], or proteins that inactivate AMPs [34]. Collectively, these adaptations allow *C*. *albicans* to successfully transition from a harmless colonizer to an invasive pathogen [22,35,36,37]. Similar mechanisms occur in other opportunistic fungi such as *Aspergillus* spp. and *Cryptococcus* spp. [34,38] (Figure 1).

Finally, regarding microbial disruption, several studies in mice have correlated mycobiota dysbiosis with various disorders [13,25]. In these studies, dysbiosis is induced by administering antibiotics or antimycotics to disrupt the bacteriota or the mycobiota, respectively. Firstly, when bacterial dysbiosis occurs, fungi such as *Candida* spp. can occupy the newly available niche and colonize this surface. Secondly, disruption of fungal communities through some antimycotic treatments has been associated with the growth of filamentous fungi since these treatments mainly target certain fungi, especially yeast like *Candida* spp., hence, they leave space for more resistant filamentous species to grow [13]. Ultimately, the altered composition of fungi may interact with human components negatively affecting human health. For example, the hyphal form of *C*. *albicans* interacts with intestinal epithelial cells (IECs) producing damage via the candidalysin toxin and the filamentous fungi expansion develops a specific immune environment that worsens allergic airway disease. Moreover, the immune response directed against the specific altered fungal species can cross-react to other fungi, increasing antifungal antibodies and exacerbating the inflammatory condition. The latter occurs when the protective response against *C*. *albicans* cross-reacts against *Aspergillus fumigatus*, an airborne fungi, causing pulmonary inflammatory disease [13,39,40].

## 3. The Interplay Between the Immune System and Fungi

The immune system is a highly sophisticated defense mechanism composed of diverse specialized cells, tissues, and molecules that defend the body from pathogens, harmful substances, and cellular alterations that could lead to cancer. It encompasses both innate immunity, which provides a rapid but non-specific response, and adaptive immunity, which generates specific and long-lasting responses through immunological memory. When it comes to fungal communities, most fungi present in the human body are commensal. In this context, the immune response plays a crucial role in establishing tolerance and regulating the delicate interplay between the host and these fungi, as even slight disruptions can lead to disease [1,41,42,43].

**Figure 2:**
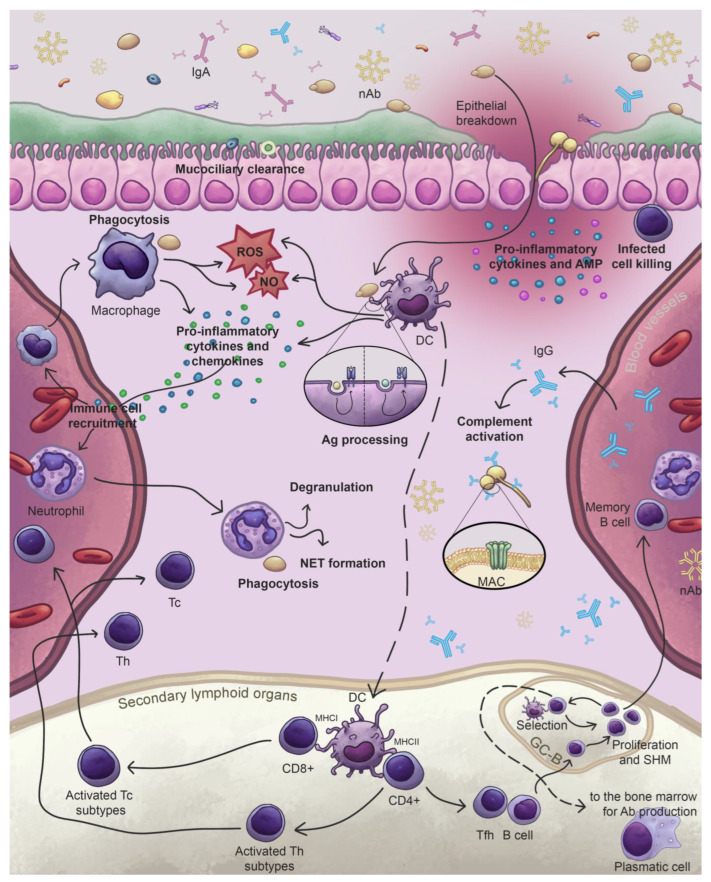
Key defense events against fungal infection. ROS: reactive oxygen species; NO: nitric oxide; AMPs: antimicrobial peptides; DCs: dendritic cell; Tc: cytotoxic T cell; Tc0: naïve cytotoxic T cell; Th: T helper cell; Th0: naïve helper T cell; nAbs: natural antibodies; IgG: Immunoglobulin G; IgA: Immunoglobulin A; GC-B: germinal center B cell; NETs: neutrophil extracellular traps.

Overall, the innate immune system is the first line of defense. It provides an immediate and non-specific response, relying on physical and physiological barriers, small molecules such antimicrobial peptides (AMPs) and cytokines, and innate immune cells like macrophages, neutrophils, and dendritic cells (DCs) [1,44]. These components rapidly detect the pathogen-associated molecular patterns (PAMPs) through the pattern recognition receptors (PRRs) and recruit additional immune cells to kill the microorganism and activate the adaptive immune system [41,45,46,47]. The adaptive immune system, in contrast, provides a highly specific response by recognizing and targeting specific antigenic determinants. It includes cellular immunity, mediated by T cells that kill infected cells directly (CD8+ T lymphocytes), or activate other immune cells and secrete cytokines to orchestrate immune responses (CD4+ T lymphocytes). Additionally, it involves humoral immunity, mediated by antibodies, which are immunoglobulins (Igs) secreted by B cells. These molecules bind specifically to antigens, neutralize pathogens, and promote their clearance through mechanisms such as opsonization and activation of the complement system [1,48] (Figure 2).

Traditionally, the innate and adaptive cellular immune systems have been considered the primary defenses against fungal infections [27,45,49,50]. This perspective was likely influenced by early studies, which suggested that antibodies were not crucial for fungal clearance, as patients with agammaglobulinemia and B cell deficiency often maintain normal antifungal immunity despite the absence of antibodies. However, some studies indicate that B cell–deficient mice are more susceptible to fatal fungal infections, and certain patients with agammaglobulinemia caused by specific mutations develop severe fungal infections [51,52,53]. Additionally, other research [54] has demonstrated that systemic antibodies generated in response to the mycobiota, particularly through the gastrointestinal route, play a protective role in safeguarding the host from lethal systemic fungal infections, regardless of their intestinal or non-intestinal origin. Notably, restoring systemic antifungal IgG targeting intestinal *C*. *albicans* provided significant protection, further supporting the critical role of antibody-mediated immunity in controlling fungal dissemination. Consequently, recent research has begun to acknowledge the role of humoral immunity in combating fungal pathogens, emphasizing that effective protection relies on the coordination of innate, cellular, and humoral responses [50,55,56]. To further explore this, the following section will examine the significance of the antibody response against fungi.

### 3.1. Activation of Adaptive Immunity

Fungi recognition triggers the processing of fungal proteins into small antigenic peptides, which are then combined with major histocompatibility complex (MHC) molecules. During antigen processing, DCs migrate to secondary lymphoid organs, where they present the processed antigen via MHC molecules to naïve T lymphocytes—MHCI for CD8+ lymphocytes and MHCII for CD4+ lymphocytes. This activates the naïve T cells through the specific binding of their T cell receptors (TCRs) to the presented antigen, triggering their differentiation into different T cell subsets depending on the activated cell and the present cytokines in the microenvironment [1,27,41,57,58]. After differentiation, they migrate to the site of infection in order to eliminate the pathogen when they recognize again the antigen presented in the respective MHC [1,27,41]. Specifically, MHC-I is expressed on all nucleated cells to present endogenous antigens to cytotoxic CD8+ T lymphocytes, while MHC-II is restricted to antigen-presenting cells (APCs), and is usually used to present exogenous antigens to CD4+ lymphocytes [1,27,41,57,58] (Figure 2).

Activated CD4+T cells secrete distinct cytokines depending on the specific subtype, such as Th1, Th2, Th17, regulatory T (Treg cells), and follicular T helper (Tfh) [1,27,41]. Specifically, when interleukin (IL)-6, IL-12, and IL-21 are produced during cells activation, these T cells would differentiate into Tfh cells. Then, the Tfh cells release IL-4, IL-10, and IL-21 cytokines, which induce the formation of germinal centers, the transformation of B cells into activated plasma cells, the production of antibodies with different isotypes (IgM, IgG, IgA, IgD, and IgE), and the production of memory B cells [1,59]. Generation of high-specificity IgG is often driven by the expansion of germinal center B (GC-B) cells, which undergo multiple cycles of somatic hypermutation (SHM) and selection that enhance antibody affinity and titers [54]. Among the five antibody isotypes, IgG is the most abundant in blood and IgA in mucous membranes [60]. B cells stimulated by this way differentiate into plasma cells, which are responsible for producing antigen-specific antibodies [44,60].

However, there are other group of antibodies, called natural antibodies (nAbs), which are produced by other different populations of B cells. They do not need the help of Thf lymphocytes and produce antibodies in a few days, which are less specific, but contribute to early immune protection. nAbs are mainly polyreactive IgM, exhibit low to medium affinity, and are often germ-line encoded. Additionally, they enhance innate immune responses, such as promoting neutrophil-mediated phagocytosis, and are essential in defending against fungal infections like aspergillosis [44,56,60].

#### 3.1.1. Adaptive Humoral Immunity Against Fungal Infections

Antibodies generated against the fungal pathogen responsible for the infection are typically directed at peptides, glycoproteins, glycolipids, and polysaccharides, many of which are integral components of the fungal cell wall. Consequently, as most antifungal antibodies target the cell wall, they influence its development, remodeling, and dynamic processes [41,56]. Likewise, the mechanisms to control fungal infections are different depending on the type of fungal species, the specificity of the epitope, and the site of infection [41,56]. Hence, the antibodies may protect the organisms from fungal infection by neutralizing fungi and their antigens; suppressing fungal growth; activating innate immune cells; altering gene expression, signaling, and lipid metabolisms; inducing iron deprivation; reducing polysaccharide release and biofilm formation; triggering fungal opsonization (enhancing immune cells’ ability to phagocyte pathogens); inducing fungal phagocytosis; activating the complement system; and activating antibody-dependent cell toxicity [51,60,61].

Among them, nAbs have been described as crucial effectors against early fungal infections [56,60,62], and recognize conserved fungal cell wall components, including β-glucan and chitin, which have remained unchanged throughout evolution. They also play a role in DCs migration and influence the generation of Th cells by directing DCs to recognize fungal antigens, facilitating their migration to lymph nodes where they present the antigens to T helper cells, activating a targeted adaptive immune response [62,63]. Thus, their importance relies on their broad reactivity to detect conserved components of the cell wall, which provides a rapid early defense despite their low affinity. Additionally, they present the capability to initiate apoptosis, promote T cell proliferation, activate complement, opsonize antigens, enhance antigenicity, direct antigens to lymph nodes, facilitate Fc receptor-mediated phagocytosis, function as adjuvants for CD8+ T cell responses, and support DC differentiation and maturation [63].

#### 3.1.2. Humoral Immunity and Commensal Fungi

Although the presence of antibodies is often associated with disease, they are not only produced against pathogenic fungi but also anti-commensal fungi response is generated as protective in healthy individuals [54,64]. Typically, the host immune system establishes tolerance to commensal fungi, maintaining a balanced immune–fungal interaction. In a study conducted by Doron et al. (2021) [54], the gut mycobiota and its antibody binding profiles were analyzed in healthy individuals. The results revealed that fungi coated with IgG, either alone (IgG^+^IgA^−^) or in combination with IgA (IgG^+^IgA^+^), were predominantly *C. albicans*. In contrast, fungi lacking both IgG and IgA (IgG^−^IgA^−^) were primarily skin- and food-associated species, such as *Malassezia restricta* and *S. cerevisiae*, which rarely cause gastrointestinal infections in humans. They concluded that IgG has preferential specificity to commensal *Candida* spp., contributing to immune homeostasis in healthy individuals and protection against invasive fungal infections, as evidenced by the selective IgG coating of *Candida* spp. in the gut, while other non-pathogenic fungi remained unrecognized, highlighting a targeted immune surveillance mechanism that balances commensalism and host defense.

Another study performed by Moreno-Sabater et al. [64] developed the Fungi-Flow method, a flow cytometry analysis combined that characterizes IgG response to commensal and environmental fungi in healthy donor cohorts. The study observed robust IgG responses to fungi such as *Penicillium* spp. and *Malassezia* spp., often associated with specific conditions, while minimal responses were detected against *Saccharomyces* spp., suggesting the presence of immunological tolerance mechanisms. Furthermore, the findings emphasized the significant impact of fungal ecosystem diversity on the intensity and variability of IgG responses, highlighting the potential for imbalances in fungal composition or immune responses to contribute to the development of diseases such as inflammatory bowel disease (IBD) and other immunopathologies.

Nevertheless, the systemic antibodies against commensal fungi can be disturbed, altering the mycobiota composition. This dysbiosis has been recognized as a crucial factor in immunopathologies that are not primarily caused by fungi such as cystic fibrosis or IBD [5,13,64,65]. Similarly, changes in the mycobiota can also disrupt the immune balance, shaping host immunity even in the absence of disease. Notably, *C*. *albicans* colonization in the gut has been shown to induce trained immunity, a phenomenon where innate immune cells develop memory-like properties, enhancing immune responses against subsequent fungal and bacterial infections. However, while *C*. *albicans* can train immunity, they can also contribute to immune-driven inflammation. Hence, imbalances in this relationship have been associated with the onset and exacerbation of multiple inflammatory disorders [13,25,66,67,68].

For instance, altered Schaedler flora (ASF) mice colonized by *C. albicans* present a high systemic IgG level, whereas ASF mice colonized by *S. cerevisiae* generated a limited IgG response. Consequently, specific fungi are suggested to drive immune responses, such as *C*. *albicans*, by modulating GC-B cell expansion in the spleen. Hence, it is suggested that *Candida* spp. presence can significantly influence and modulate the host’s immune response.

### 3.2. Antibodies as Biomarkers

Biomarkers are defined as molecules, parameters, structures, or processes that can be objectively measured and evaluated to obtain objective information about a patient’s condition, allowing the differentiation of physiological status from pathological ones. Additionally, a reliable biomarker should be reproducible, precise, easy to interpret, cost-effective, highly sensitive, and specific, and provide additional insights beyond clinical variables. It should be noted that a biomarker does not need to be studied alone, but it can be studied along with other biomarkers [69].

Consequently, since dysbiosis in the microbiota can both cause and result from diseases that are not primarily fungal and considering that the humoral response targets both pathogenic and commensal fungi, antibodies could serve as indicators of the mycobiota–immune response balance and are proposed as biomarkers for diagnosing and predicting certain diseases [5,13,64,65,66]. Furthermore, IgGs are present in high concentrations in serum and can stay stable for several years at −20 °C or −80 °C, making serological samples a valuable resource for studying antibody responses. Moreover, serum is the choice sample to collect for many studies for its richness in biological information; hence, it is routinely collected in clinical practice and its use does not interfere with standard protocols. Additionally, compared to other sampling techniques, it is a less invasive method for assessing immune responses, as it is easy to store and may be collected without additives, among other advantages [70,71,72,73,74,75].

Traditional methods for studying the mycobiota are often limited. Fungal identification has historically relied on culture-based techniques, which are inefficient due to the low cultivability of many fungal species, time-consuming techniques, and possible misidentification [66]. More recent approaches, such as next-generation sequencing (NGS) or Matrix Assisted Laser Desorption Ionisation—Time of Flight Mass Spectrometry (MALDI-TOF MS), offer higher sensitivity but depend on pre-existing genomic and protein spectrum databases for fungal identification. However, these databases are predominantly composed of bacterial genomes, making fungal detection challenging [66]. Moreover, both culture methods and NGS only provide information about the composition of the mycobiota without offering insight into the host’s immune response, which, as it has been seen, is equally important to study in conjunction.

In contrast, measuring IgG responses may serve as a more reliable indicator of fungal exposure than direct fungal detection, as IgG antibodies against *C*. *albicans* have been shown to persist beyond the initial colonization event, even in the absence of detectable live fungi in multiple organs [54]. This highlights the potential of antibody-based studies to bridge the gap between fungal presence and the immune system’s long-term response, providing a more comprehensive understanding of host–fungal interactions.

Finally, a variety of established techniques for antibody analysis are available, including enzyme-linked immune sorbent assay (ELISA) [76,77,78,79], dot immunobinding assay (DIA) [80,81,82], Western blotting (WB) [76,83], immunofluorescence [76], immunochromatography [84], and Luminex assays [85]. As such, it would be feasible to adapt these methods to specifically capture and detect antifungal antibodies.

## 4. Antifungal Antibodies as Tools for Diagnosis and Predicting Chronic Immune-Mediated Inflammatory Non-Communicable Disease

Fungal antigens and their corresponding immune responses are increasingly recognized as potential biomarkers for diagnosing and predicting the course of chronic immune-mediated inflammatory non-communicable diseases (IMID), such as Chron’s disease (CD), ulcerative colitis (UC), rheumatoid arthritis, Sjögren’s syndrome, asthma, multiple sclerosis (MS), or systemic lupus [86,87,88]. In fact, in some of these diseases, antifungal antibody titer analysis provides valuable insights into disease pathogenesis and immune dysregulation, helping to identify at-risk individuals and guide therapeutic interventions.

### 4.1. Current Applications

Currently, the most important studied antifungal antibodies as biomarkers in IMID are anti-*Saccharomyces cerevisiae* antibodies (ASCAs) to differentiate CD from UC among IBD [13,89,90,91,92]. Indeed, ASCA levels are considered to be a prognostic marker for CD, as their levels tend to increase at the beginning of the disease and decrease after successful therapy or surgical resection. ASCA status may also be linked to disease relapse, highlighting its potential role in predicting the course of the disease. Additionally, ASCAs have been associated with earlier disease onset, ileal involvement, fibrostenosis, and a higher probability of requiring surgical resection. Furthermore, ASCA positivity in children with CD may help identify those who could benefit from earlier introduction of biological therapies to manage inflammation more effectively [90,92].

However, ASCAs are currently being detected in many other human diseases such as autoimmune liver disease (AILD) [13,93,94], primary sclerosing cholangitis (PSC) [13,93,95], primary biliary cirrhosis (PBC) [13,93,96], and Behçet’s disease (BD) [89,97], among other immune-mediated diseases [13,89,90,91]. Moreover, the presence of ASCAs in neurological diseases, such as Parkinson’s disease (PD) [90], depression [90,98], and bipolarity [90,98], is linked to the role of the gut–brain axis in human immunity, influencing both health and disease.

ASCAs are antibodies directed against the fungal cell wall component oligomannose, which is present in *S*. *cerevisiae*, commonly known as baker’s or brewer’s yeast, as well as in *C. albicans*. One hypothesis explaining the presence of ASCA IgG and IgA in humans is that *S*. *cerevisiae* has long been used in the food industry for fermentation, leading to frequent human exposure and its integration as part of the healthy gut mycobiota. Consequently, this exposure may trigger an immune response [89,92]. However, another hypothesis derived from the link between ASCA and fungal dysbiosis suggests that changes in the gut’s fungal composition, particularly *Candida* spp. overgrowth, may trigger immune responses that drive ASCA production [90]. This idea is further supported by the fact that ASCA epitopes are also expressed in *C*. *albicans*, suggesting the possibility of a cross-reactive immune response between both fungi in the intestine. This cross-reactivity has been observed not only with the abundant *C*. *albicans* but also with less abundant fungal genera such as *Aspergillus* spp. *s* and *Malassezia* spp.

Furthermore, given the structural similarity between microbial glycans from fungi, bacteria, and viruses and host glycoconjugates, it has been proposed that glycan mimicry may contribute to the development of immune-mediated disease. In this context, ASCAs, initially induced by exposure to fungi like *S*. *cerevisiae* or *C*. *albicans*, could cross-react with similar structures in other microorganisms and, in some cases, with glycans present in host cells. This phenomenon of molecular mimicry could lead to the generation of autoantibodies against self-antigens, triggering chronic inflammatory responses and contributing to the development of diseases [90,99]. Consequently, ASCAs are believed to also target human molecules, which strengthens the idea that, although initially induced by microbial exposure, ASCAs could persist as an auto-antibody due to molecular mimicry involving host glycoproteins. This autoimmune response could contribute to the chronic inflammation seen in CD [90].

While ASCA remains the most studied antifungal antibody, research has expanded to include other fungal biomarkers such as the anti-glycan carbohydrate antibodies anti-laminaribioside antibodies (ALCAs), anti-chitobioside antibodies (ACCAs), and anti-mannobioside IgG (AMCA), which also play a role in CD, with ASCAs and ALCAs being the most relevant biomarkers for CD [90,100,101,102,103].

However, growing evidence suggests that IgG responses against fungi extend beyond IBD, influencing a wide range of neurological, autoimmune, and inflammatory diseases (Table 1).

### 4.2. Future Potential

Recent studies have linked dysbiosis to certain diseases, leading to further research on antifungal antibodies as potential biomarkers. Their presence in blood may indicate an immune response to fungal overgrowth, which could play a role in disease development. This section explores their potential applications in neurological, psychiatric, and neurodegenerative disorders and cancer [104,105,106,107,108,109,110] (Table 1).

This has been explored in conditions such as autism spectrum disorder (ASD), where *Candida* species overgrowth has been observed, and IgG titers may serve as potential biomarkers [104,111]. Hughes and Ashwood [104] carried out a study in which they observed an increase in IgGs against *C*. *albicans* in children with ASD compared to typically developing children, probably caused by fungal overgrowth in their gastrointestinal tract. While the exact cause of the dysbiosis remains unclear, this overgrowth may contribute to both gastrointestinal and behavioral symptoms in ASD. These findings suggest that antifungal antibodies could serve as potential biomarkers for ASD, reflecting an altered immune response to fungal overgrowth.

On the other hand, although the etiology of psychiatric disorders is primarily influenced by genetic and environmental factors, recent studies have highlighted the role of the immune system. In particular, exposure to certain infectious microorganisms during neurodevelopment can activate immune responses that may lead to damage in the central nervous system, influencing the development of psychiatric disorders [105]. Severance et al. [105,106,107] observed higher titers of antibodies targeting *S*. *cerevisiae* and *C*. *albicans* in bipolar (BD) and schizophrenia (SCZ) disorders compared to people without a history of psychiatric disorders. Particularly, in BD females, *Candida* antibodies are related to lower cognition scores, while in males with SCZ, *C*. *albicans* showed a direct and strong correlation with antibody titers and has been identified as a potential risk factor for the disorder, independent of confounding factors such as age, race, or medication [105]. Regarding ASCA IgGs, they were measured as indicators of gastrointestinal inflammation in schizophrenia and bipolarity, but they do not directly imply a causal role in the disorder [106,107]. These findings suggest a potential link between fungal exposure, immune response, and psychiatric disorders.

Elevated IgG antibodies against the mycobiota have also been noted in neurodegenerative diseases, such as Alzheimer’s disease (AD) [108] or PD [109]. In AD, antibodies against several *Candida* species were found to be increased compared to healthy subjects. Furthermore, next-generation sequencing has revealed the prevalence of specific fungal genera, including *Alternaria* spp., *Botrytis* spp., *Candida* spp., and *Malassezia* spp., in the brain tissue of AD patients. It is hypothesized that infection leads to an immune response that contributes to amyloid deposition, neuroinflammation, and vascular damage, although the direct cause of AD remains unclear [108,112]. Elevated ASCAs has been observed in serum samples of de novo PD [109]. Moreover, due to the association between seborrheic dermatitis (SD) and PD, the investigation of the presence of *Malassezia*, the fungus responsible for SD, has been suggested in relation to PD [113]. Upon confirming the relationship between *Malassezia* spp. and PD, anti-*Malassezia* spp. antibody titers can be examined to assess their potential as a clinical biomarker. Similarly, in MS the role of fungi has also been suggested as crucial in its pathogenesis. *Candida* spp. and fungal toxins have been linked to the neuroinflammation and myelin damage observed in MS [114]. In fact, Consequently, since antifungal immunity overlaps with the MS-related immune response, antifungal antibodies could also be studied during the prognosis and diagnosis of the disease. Given these findings, antifungal antibodies may serve as potential biomarkers for neurodegenerative diseases, helping to assess disease progression or even identify at-risk individuals.

Moreover, emerging evidence suggests a potential link between the mycobiota and colorectal cancer (CRC). Patients with CRC exhibit distinct fungal alterations, including an increased *Candida*/*Saccharomyces* ratio, which correlates with disease progression and metastasis [115,116]. *Candida albicans* contributes to a pro-inflammatory environment by triggering interleukin-mediated and cytokine-mediated responses and disrupting epithelial barrier integrity, promoting tumorigenesis [13,116,117]. These fungal shifts elicit immune responses, reflected in elevated antifungal antibody titers in CRC patients [118]. Given their association with disease-related dysbiosis, antifungal antibodies may serve as potential biomarkers for CRC, aiding in early detection and prognosis.

Therefore, all these studies show that antifungal antibodies hold promise as biomarkers for the diagnosis of several diseases and monitoring of their progression, but additional investigations are required. Future studies should focus on clarifying their role in disease pathophysiology and assessing their potential as clinical biomarkers through well-designed longitudinal cohort studies and case-control analyses. Additionally, randomized clinical trials could help determine their specificity, reliability, and clinical relevance in different patient populations. Establishing these aspects could improve early detection and facilitate targeted treatments.

**Table 1:** Examples of conditions where mycobiota imbalance and associated antibody production are correlated with the condition diagnosis, disease severity, or prognosis.

Type of Illnesses	Disease, Disorder or Condition	Immune and Mycobiota Imbalance	Reference
Gut disease	Crohn’s disease	Increase in ASCA, ALCA, ACCA, AMCA antibodies, often associated with severe or complicated disease Pathogenic antibodies to mannose glycan associated with IgG glycosylation signature *Candida* spp. overgrowth	[90,92,100,101,102,103]
	Colorectal cancer	Increased *Candida* spp., decreased *Saccharomyces* spp.	[115,116]
Cognitive disorders	Autism spectrum disorder	*C*. *albicans* overgrowth and increase in IgG antibodies	[90,104,111]
	Bipolar disorder	Increased antibodies against *S*. *cerevisiae* Elevated ASCA markers	[105,107]
	Schizophrenia	Increased antibodies against *C*. *albicans*: diagnostic marker in males, prognostic marker for cognitive decline in females. Elevated ASCA markers (especially in antipsychotic-naive individuals Higher *Candida* spp., *C. dubliensis*	[105,106]
	Alzheimer’s disease	Elevated antibodies against *Candida* spp. in some patients Prevalence of *Alternaria* spp., *Botrytis* spp., *Candida* spp., and *Malassezia* spp.	[108,112]
	Parkinson’s disease	Increased ASCA antibodies Presence of *Malassezia*	[109,113]
	Multiple sclerosis	Presence of *Trichosporon mucoides* and *Candida deformans*	[114,119]

## 5. Challenges and Limitations

One of the main challenges that many researchers agree on is the relative lack of studies on the mycobiota compared to the bacteriota within the human microbiota. Fungi are mostly overlooked, and when studied, the research tends to center on pathogenic fungi, particularly their infection mechanisms, immune responses, potential treatment targets, and antifungal dosage [1,120,121].

However, it is not surprising that this issue persists. The current techniques employed to study the mycobiota are predominantly based on computational methods that compare the results obtained with available databases. For instance, fungal genome sequencing (20,393 genomes) is significantly less extensive than bacterial genome sequencing (2,515,028 genomes), which can give the impression that bacteria are more relevant than fungi, when in reality, fungal presence could be more evenly distributed. The limited fungal genome database restricts the ability to fully characterize host-associated fungal communities, underscoring the need for more comprehensive sequencing efforts. Furthermore, the lack of consistent bioinformatics platforms and the limited data available from different mucosal surfaces pose significant challenges in mycobiota studies. Nevertheless, recent advances have made it possible to define core mycobiomes at various body sites and investigate how they change during inflammation [5,13,66,121,122].

Additionally, although some research groups are dedicated to studying the effects of the mycobiota on health and disease, most investigations are conducted using animal models [123,124,125,126], which have limitations in being fully extrapolated to human mycobiota interactions, as animal and human ecosystems differ significantly [64]. To address this, organ-on-a-chip studies could be developed to create more physiologically relevant models for human mycobiota research [127,128]. Likewise, due to the lower fungal burden compared to bacteria, many methods that analyze both bacteria and fungi tend to mask the effects of fungi, as the dominant bacterial presence overshadows their impact [64,121,129].

Setting aside the issue of fungi per se, the study of antibodies against fungi has not been extensively explored, as immune responses to fungi were considered irrelevant until the 1990s. The role of antibodies was not studied until after this period, when experimental methods began to show a potential involvement of antibody-mediated immunity in host defense against fungal infections [50,130]. Additionally, although some immunological tests detecting antifungal antibodies are already commercially available, they are designed for only a few species. Furthermore, selecting the optimal antigen to detect specific antibodies is challenging, especially considering the cross-reactivity observed between certain antifungal antibodies [44,131].

In summary, despite the increasing interest in the mycobiota and its implications for human health and disease, several challenges persist, including limited databases, inconsistent bioinformatics tools, and the need for more comprehensive studies. Addressing these gaps requires large-scale metagenomic and metatranscriptomic studies to improve fungal genome databases, standardized bioinformatics pipelines for mycobiota analysis, and more longitudinal human cohort studies to better understand fungal community dynamics. Additionally, clinical studies assessing antifungal antibodies as biomarkers should focus on specificity, sensitivity, and their correlation with disease progression. Overcoming these limitations could pave the way for a more holistic understanding of fungal communities and their immune interactions, opening up new avenues for diagnostics and treatment strategies (Table 2).

## 6. Future Perspective

Research on the humoral immune response to the mycobiota and its potential as a diagnostic and prognostic tool is in an emerging phase, with significant opportunities for its future development. Several key areas could drive significant advances in this field and improve the clinical application of antifungal antibodies as biomarkers of human diseases.

As mentioned above, one of the main current challenges is the lack of systematic studies on the mycobiota compared to the bacteriota. As fungal genomic and proteomic databases expand, an improvement in the ability to identify fungal species and their interaction with the immune system, as well as their interaction with other species, is expected. Emerging technologies, such as NGS and the development of more sophisticated bioinformatics platforms, will allow for a more precise analysis of fungal diversity in different tissues and their role in homeostasis or dysbiosis.

The potential of antibodies against the mycobiota as clinical biomarkers could also benefit from multiplexing in serological studies. Instead of analyzing a single antibody in isolation, the use of multiplex detection platforms could provide a more comprehensive view of the antifungal immune response in IMID, such as chronic inflammatory diseases and autoimmune, as well as in neurodegenerative disorders. Future studies should focus on evaluating the combination of immunoglobulins against different microorganisms to establish predictive serological profiles with greater specificity and sensitivity. For instance, microarray technology could be leveraged to achieve this goal, as demonstrated in previous studies that simultaneously analyze multiple analytes. Examples include the detection of IgE in dogs against various allergens [132] and the identification of human IgGs targeting membrane antigens in tissues from both animal models and humans, which serve as biomarkers for autoimmune disorders [133].

Likewise, antifungal antibodies could be integrated into early disease screening programs in at-risk populations. Their use in the detection of certain pathologies such as IBD, and psychiatric or neurodegenerative disorders, could facilitate earlier interventions and improve long-term clinical outcomes. However, before their widespread implementation, these biomarkers will need to be validated in large-scale multicenter studies and their applicability in different clinical contexts will need to be assessed.

Finally, another promising approach is the development of therapeutic strategies based on the modulation of the mycobiota. The use of probiotics and prebiotics specifically targeting the balance of the fungal community could represent a new avenue to treat diseases associated with fungal dysbiosis. Furthermore, personalizing these therapies based on the antifungal antibody profile of each patient would allow for more precise and tailored medicine for each individual. Along these lines, research on FMT to restore fungal balance is also seen as an area of growing interest.

In conclusion, the exploration of antibodies against the mycobiota as diagnostic and prognostic tools offers an expanding field of research with multiple possibilities. With technological advances in sequencing, bioinformatics, and immunological studies, it is expected that in the near future, significant progress will be made in the characterization of the mycobiota and its impact on human health (Table 2).

## Figures and Tables

**Table 2:** SWOT analysis of the mycobiota research for the diagnosis and prognosis of human diseases.

Strengths	Weaknesses
Emerging interest in the mycobiota Technological advancement in sequencing and bioinformatics Potential of antifungal antibodies as biomarkers Opportunities for personalized medicine	Limited mycobiota research and data Insufficient fungal databases Cross-reactivity in antibody detection Animal model limitations
**Opportunities**	**Threats**
Expansion of fungal databases Multiplex detection for better diagnosis Early disease screening using antibodies Targeted therapies for fungal dysbiosis	Lack of large-scale validation studies Bacterial dominance masking fungal effects Regulatory hurdles for clinical implementation

## Abbreviations

The following abbreviations are used in this manuscript:

ACCAs	Anti-chitobioside antibodies
AD	Alzheimer’s disease
AILD	Autoimmune liver disease
ALCAs	anti-laminaribioside antibodies
AMCA	anti-mannobioside IgG
AMPs	Antimicrobial peptides
APCs	Antigen-presenting cells
ASCAs	Anti-Saccharomyces cerevisiae antibodies
ASD	Autism spectrum disorder
ASF mice	Altered Schaedler flora mice
BD	Behçet’s disease
BD	Bipolar disorder
CD	Crohn’s disease
CRC	Colorectal cancer
DCs	Dendritic cells
DIA	Dot immunobinding assay
ELISA	Enzyme-linked immune sorbent assay
FMT	Fecal microbiota transplant
GC-B	Germinal center B
IBD	Inflammatory bowel disease
IECs	Intestinal epithelial cells
Igs	Immunoglobulins
IL	Interleukin
IMID	Immune-mediated inflammatory disease
MAC	Membrane attack complex
MALDI-TOF MS	Matrix Assisted Laser Desorption Ionisation—Time of Flight Mass Spectrometry
MHC	Major histocompatibility complex
MS	Multiple sclerosis
nAbs	Natural antibodies
NGS	Next-generation sequencing
PAMPs	Pathogen-associated molecular patterns
PBC	Primary biliary cirrhosis
PD	Parkinson’s disease
PMNs	Polymorphonuclear leukocytes
PRRs	Pattern recognition receptors
PSC	Primary sclerosing cholangitis
ROS	Reactive oxygen species
SCZ	Schizophrenia
SD	Seborrheic dermatitis
SHM	Somatic hypermutation
SWOT	Strengths, weaknesses, opportunities, and threats analysis
Tc cells	Cytotoxic T cells
TCRs	T cell receptors
Tfh cells	Follicular helper T cells
Th cells	Helper T cells
Treg cells	Regulatory T cells
UC	Ulcerative Colitis
WB	Western blotting
